# Supplementary material for: Unveiling Shadows: Investigating women’s experience of intimate partner violence in Ghana through the lens of the 2022 Demographic and Health Survey
Source: PLoS One. 2024 Aug 29;19(8):e0309564. doi: 10.1371/journal.pone.0309564 (PMC11361440; doi:10.1371/journal.pone.0309564)
Supplement: Attachment — Submitted filename: PONE-D-24-14998 (1).pdf [file pone.0309564.s001.pdf]

PLOS ONE

Unveiling Shadows: Investigating women’s experience of intimate partner violence in Ghana through the lens of the 2022 Demographic and Health Survey

--Manuscript Draft--

**Manuscript Number:**	PONE-D-24-14998
**Article Type:**	Research Article
**Full Title:**	Unveiling Shadows: Investigating women’s experience of intimate partner violence in Ghana through the lens of the 2022 Demographic and Health Survey
**Short Title:**	Intimate partner violence against women in Ghana
**Corresponding Author:**	Joshua Okyere<br>University of Cape Coast<br>Tema, Accra GHANA
**Keywords:**	Intimate Partner; violence; women; Social Norms
**Abstract:**	Introduction: Intimate partner violence (IPV) is a significant public health issue, predominantly among women in sub-Saharan Africa, including Ghana. Existing evidence indicates high rates of IPV perpetration and its associated adverse health outcomes. Despite previous studies, reliance on old data underscores the need for current, nationally representative data to inform policy-making and interventions. Therefore, this study utilizes the 2022 Ghana Demographic and Health Survey (GDHS) to examine IPV experiences and associated factors, aiming to provide updated insights for effective IPV mitigation strategies in Ghana<br>Methods: This is a cross-sectional study of a weighted sample of 3,741 women between the ages of 15-49 years old from the 2022 GDHS. Binary logistic regression analysis was used in predicting the outcome.<br>Results: The study found that 36.4 percent of women in Ghana had experienced some form of IPV, primarily emotional violence (31.5%), physical violence (17.3%), and sexual violence (7.6%). Women with higher levels of education had a significantly reduced risk of 60% of experiencing some form of IPV than those with no formal education. Partner domineering behaviours, such as women whose partners often get jealous for seeing them talk with other men [adjusted OR:1.76, 95%CI:1.25,2.48], accusing them of unfaithfulness [adjusted OR:2.59, 95%CI:1.03,2.46], not permitting them to meet female friends [adjusted OR:1.1.59, 95%CI:1.03,2.46], and limiting their contact with family [adjusted OR:5.75, 95%CI:2.27,13.42], were more likely to experience at least one form of IPV. Similarly, women who justified or endorsed wife beating had a higher likelihood [aOR=1.57, 95%CI=1.22,2.02] of experiencing at least one form of IPV than those who did not endorse such behaviour.<br>Conclusion: Identifying educational attainment, partner dominance, and violence endorsement as IPV predictors underscore targeted interventions. Promoting women's education bolsters empowerment and IPV prevention. Addressing dominance through education, counselling, and legal frameworks is crucial for fostering safer relationships and challenging violence normalization.
**Order of Authors:**	Kwamena Sekyi Dickson
	Castro Ayebeng
	Joshua Okyere
**Additional Information:**	
**Question**	**Response**
**Financial Disclosure**<br><br>Enter a financial disclosure statement that describes the sources of funding for the work included in this submission. Review the submission guidelines for detailed	The author(s) received no specific funding for this work.

Powered by Editorial Manager® and ProduXion Manager® from Aries Systems Corporation

requirements. View published research articles from *PLOS ONE* for specific examples.<br><br>This statement is required for submission and **will appear in the published article** if the submission is accepted. Please make sure it is accurate.<br><br>**Funded studies**<br>Enter a statement with the following details:<br>• Initials of the authors who received each award<br>• Grant numbers awarded to each author<br>• The full name of each funder<br>• URL of each funder website<br>• Did the sponsors or funders play any role in the study design, data collection and analysis, decision to publish, or preparation of the manuscript?<br><br>Did you receive funding for this work?	
**Competing Interests**<br><br>Use the instructions below to enter a competing interest statement for this submission. On behalf of all authors, disclose any competing interests that could be perceived to bias this work—acknowledging all financial support and any other relevant financial or non-financial competing interests.<br><br>This statement is required for submission and **will appear in the published article** if the submission is accepted. Please make sure it is accurate and that any funding sources listed in your Funding Information later in the submission form are also declared in your Financial Disclosure statement.<br><br>View published research articles from *PLOS ONE* for specific examples.	The authors have declared that no competing interests exist.

Powered by Editorial Manager® and ProduXion Manager® from Aries Systems Corporation

**NO authors have competing interests**<br><br>Enter: *The authors have declared that no competing interests exist.*<br><br>**Authors with competing interests**<br><br>Enter competing interest details beginning with this statement:<br><br>*I have read the journal's policy and the authors of this manuscript have the following competing interests: [insert competing interests here]*<br><br>* typeset	
**Ethics Statement**<br><br>Enter an ethics statement for this submission. This statement is required if the study involved:<br><br>• Human participants<br>• Human specimens or tissue<br>• Vertebrate animals or cephalopods<br>• Vertebrate embryos or tissues<br>• Field research<br><br>Write "N/A" if the submission does not require an ethics statement.<br><br>General guidance is provided below. Consult the submission guidelines for detailed instructions. **Make sure that all information entered here is included in the Methods section of the manuscript.**	We did not need to seek ethical clearance because the DHS dataset we used is publicly available. We obtained the datasets from the DHS Program after completing the necessary registration and getting approval for their use. We followed all the ethical guidelines that pertain to using secondary datasets in research publications. You can find detailed information about how we used the DHS data and the ethical standards we followed at this link: http://goo.gl/ny8T6X.

**Format for specific study types**<br><br>**Human Subject Research (involving human participants and/or tissue)**<br>• Give the name of the institutional review board or ethics committee that approved the study<br>• Include the approval number and/or a statement indicating approval of this research<br>• Indicate the form of consent obtained (written/oral) or the reason that consent was not obtained (e.g. the data were analyzed anonymously)<br><br>**Animal Research (involving vertebrate animals, embryos or tissues)**<br>• Provide the name of the Institutional Animal Care and Use Committee (IACUC) or other relevant ethics board that reviewed the study protocol, and indicate whether they approved this research or granted a formal waiver of ethical approval<br>• Include an approval number if one was obtained<br>• If the study involved *non-human primates*, add *additional details* about animal welfare and steps taken to ameliorate suffering<br>• If anesthesia, euthanasia, or any kind of animal sacrifice is part of the study, include briefly which substances and/or methods were applied<br><br>**Field Research**<br><br>Include the following details if this study involves the collection of plant, animal, or other materials from a natural setting:<br>• Field permit number<br>• Name of the institution or relevant body that granted permission	
**Data Availability**<br><br>Authors are required to make all data underlying the findings described fully available, without restriction, and from the time of publication. PLOS allows rare exceptions to address legal and ethical concerns. See the PLOS Data Policy and FAQ for detailed information.	Yes - all data are fully available without restriction

A Data Availability Statement describing where the data can be found is required at submission. Your answers to this question constitute the Data Availability Statement and **will be published in the article**, if accepted.<br><br>**Important:** Stating 'data available on request from the author' is not sufficient. If your data are only available upon request, select 'No' for the first question and explain your exceptional situation in the text box.<br><br>Do the authors confirm that all data underlying the findings described in their manuscript are fully available without restriction?	
**Describe where the data may be found in full sentences. If you are copying our sample text, replace any instances of XXX with the appropriate details.**<br><br>• If the data are **held or will be held in a public repository**, include URLs, accession numbers or DOIs. If this information will only be available after acceptance, indicate this by ticking the box below. For example: *All XXX files are available from the XXX database (accession number(s) XXX, XXX.).*<br>• If the data are all contained **within the manuscript and/or Supporting Information files**, enter the following: *All relevant data are within the manuscript and its Supporting Information files.*<br>• If neither of these applies but you are able to provide **details of access elsewhere**, with or without limitations, please do so. For example:<br><br>*Data cannot be shared publicly because of [XXX]. Data are available from the XXX Institutional Data Access / Ethics Committee (contact via XXX) for researchers who meet the criteria for access to confidential data.*<br><br>*The data underlying the results presented in the study are available from (include the name of the third party*	The datasets generated and/or analysed during the current study are available in the Measure DHS repository: http://dhsprogram.com/data/available-datasets.cfm.

Powered by Editorial Manager® and ProduXion Manager® from Aries Systems Corporation

and contact information or URL).<br>• This text is appropriate if the data are owned by a third party and authors do not have permission to share the data.<br><br>* typeset	
Additional data availability information:	

Powered by Editorial Manager® and ProduXion Manager® from Aries Systems Corporation

Manuscript

Click here to access/download;Manuscript;UAPS.Intimate Partner Violence among women Ghana.docx

Unveiling Shadows: Investigating women's experience of intimate partner violence in Ghana through the lens of the 2022 Demographic and Health Survey

Kwamena Sekyi Dickson[1], Castro Ayebeng[1], and Joshua Okyere[1,2*]

1. Department of Population and Health, University of Cape Coast, Cape Coast, Ghana.
2. School of Nursing and Midwifery, College of Health Sciences, Kwame Nkrumah University of Science and Technology, Kumasi, Ghana

Corresponding author*

Emails:

KSD: kwamena.dickson@ucc.edu.gh

CA: castro.ayebeng@stu.ucc.edu.gh

JO: joshuaokyere54@gmail.com

Abstract

Introduction: Intimate partner violence (IPV) is a significant public health issue, predominantly among women in sub-Saharan Africa, including Ghana. Existing evidence indicates high rates of IPV perpetration and its associated adverse health outcomes. Despite previous studies, reliance on old data underscores the need for current, nationally representative data to inform policy-making and interventions. Therefore, this study utilizes the 2022 Ghana Demographic and Health Survey (GDHS) to examine IPV experiences and associated factors, aiming to provide updated insights for effective IPV mitigation strategies in Ghana

Methods: This is a cross-sectional study of a weighted sample of 3,741 women between the ages of 15-49 years old from the 2022 GDHS. Binary logistic regression analysis was used in predicting the outcome.

Results: The study found that 36.4 percent of women in Ghana had experienced some form of IPV, primarily emotional violence (31.5%), physical violence (17.3%), and sexual violence (7.6%). Women with higher levels of education had a significantly reduced risk of 60% of experiencing some form of IPV than those with no formal education. Partner domineering behaviours, such as women whose partners often get jealous for seeing them talk with other men [adjusted OR:1.76, 95%CI:1.25,2.48], accusing them of unfaithfulness [adjusted OR:2.59, 95%CI:1.03,2.46], not permitting them to meet female friends [adjusted OR:1.1.59, 95%CI:1.03,2.46], and limiting their contact with family [adjusted OR:5.75, 95%CI:2.27,13.42], were more likely to experience at least one form of IPV. Similarly, women who justified or endorsed wife beating had a higher likelihood [aOR=1.57, 95%CI=1.22,2.02] of experiencing at least one form of IPV than those who did not endorse such behaviour.

Conclusion: Identifying educational attainment, partner dominance, and violence endorsement as IPV predictors underscore targeted interventions. Promoting women's education bolsters empowerment and IPV prevention. Addressing dominance through education, counselling, and legal frameworks is crucial for fostering safer relationships and challenging violence normalization.

Keywords: Intimate Partner; Violence; Women; Social Norms

Background

Intimate partner violence (IPV) – that is, any act perpetrated by an intimate partner to cause physical, emotional and sexual harm – is considered a serious public health concern and an infringement on human rights [1]. Globally, 1 in 3 women are victims of IPV [2]. In sub-Saharan Africa (SSA), approximately 33% of women experience IPV at some point in their lifetime [3]. These statistics highlight the urgency for all countries, particularly those located in SSA to work towards the elimination of IPV.

Evidence from Ghana [4] indicates that 28.2 percent of women justify their partners' perpetuation of IPV. This is worrying given that IPV is known to be associated with adverse physical, social and sexual health outcomes. For instance, women who experience sexual violence are 1.11 times more likely to engage in multiple high-risk fertility behaviours [5]. Furthermore, IPV perpetuation has been linked to higher risk of subsequent violence victimization, psychological distress, and unplanned pregnancies [6,7]. One integrative review [8] has reported that women who experience IPV are more likely to experience cardiovascular symptoms.

Indeed, there is extant literature on IPV in Ghana. However, most of these studies were either based on the 2014 Ghana Demographic and Health Survey (GDHS) [4,9,10] or qualitative research approaches [11,12]. However, given the significant time lapse between 2014 and 2022, relying solely on the findings of the 2014 GDHS may not accurately capture the current landscape of IPV experienced by women in Ghana. This underscores the critical need for more recent, nationally representative data to assess the prevalence of IPV, its various typologies, and the factors associated with it. Consequently, this study seeks to address this gap by utilizing the 2022 GDHS to examine women's experiences of IPV in Ghana and explore the factors contributing to it. By doing so, the study aims to provide up-to-date insights that can inform policy-making, intervention strategies, and support services aimed at addressing and mitigating IPV in Ghana.

Methods

Data source

Data source and design

The analysis utilized data from the 2022 Ghana Demographic and Health Survey (GDHS), specifically drawing from the individual recode file (IR). The GDHS is part of a larger initiative spanning 85 low-and-middle-income countries (LMICs) [13]. The primary objective of the 2022 GDHS was to furnish current estimations of fundamental demographic and health indicators. To achieve this goal, a meticulously designed two-staged sampling strategy was implemented, resulting in a stratified representative sample comprising 18,450 households distributed across 618 clusters. This method ensured thorough representation at the national level, encompassing both urban and rural areas, and within each of Ghana's 16 regions [14].

During the initial stage, 618 target clusters were identified using a probability proportional to size method, considering urban and rural differentiations within each region [14]. Subsequently, an equal probability systematic random sampling approach was employed to choose the requisite number of clusters in both urban and rural settings. Moving to the second stage, subsequent to cluster selection, an exhaustive household listing and map updating process were conducted within all selected clusters, establishing a comprehensive roster of households for each cluster. From each cluster, 30 households were randomly chosen for interviews [14]. For further information on the design of the GDHS, refer to. https://www.dhsprogram.com/Methodology/Survey-Methodology.cfm

Data collection was performed using structured household and women's questionnaires administered by trained enumerators. The women's questionnaire covered various aspects, including birth history, childhood mortality, fertility preferences, child health, maternal health, and domestic violence. The analysis consisted of a weighted sample of 3,741 women between the ages 15-49 years old who were either married or cohabiting and have information on intimate partner violence considered in this study. The use of the dataset did not require ethical clearance since it was obtained from a secondary data source. Nevertheless, permission to use the dataset was obtained from the Measure DHS.

Study variables and measurements

Outcome variable

Intimate partner violence was derived from four outcome variables. These are experienced sexual violence ("yes" or "no."), emotional violence ("yes" or "no."), physical violence ("yes" or "no.") and experienced at least one form of violence ("yes" or "no.").

Explanatory variables

Multiple factors informed by theoretical and empirical literature relating to intimate partner violence [4,11,15,16] were included in the analyses as the explanatory variables. These variables include age (15-19, 20-24, 25-29, 30-34, 35-39, 40-44, 45-49), region of residence (Western, Central, Greater Accra, Volta, Eastern, Ashanti, Western North, Ahafo, Bono, Bono East, Oti, Northern, Savannah, North East, Upper East, Upper West) residence (rural and urban), level of education (no education, basic, secondary and above), wealth index (poorest, poorer, middle, richer, richest), partner domineering variables include husband/partner are jealous if respondent talks with other men (never, often, sometimes, yes, but not in the last 12 months) , husband/partner accuses respondent of unfaithfulness (never, often, sometimes, yes, but not in the last 12 months) , husband/partner does not permit respondent to meet female friends (never, often, sometimes, yes, but not in the last 12 months), husband/partner tries to limit respondent's contact with family (never, often, sometimes, yes, but not in the last 12 months), husband/partner insists on knowing where respondent (never, often, sometimes, yes, but not in the last 12 months), and justification of wife beaten (no, yes).

Data analysis

Statistical analysis was performed using Stata version 17. Both descriptive and inferential statistics were conducted. Further, univariate and multivariate analyses were conducted to explore the relationships between the explanatory variables and outcome variable. Logistic regression models were fitted using STATA regression commands to assess adjusted risk factors associated with the study outcomes, with Odds Ratios (OR) and 95% confidence intervals (CI) calculated. To account for any sampling bias from under or over-sampling of respondents in the total population, all descriptive estimates were weighted using the

individual weight variable (d005) in the dataset. To consider the complex survey design of the DHS data, the "svyset" command in Stata was employed.

Results

Prevalence of intimate partner violence

Figure 1 shows the prevalence of IPV among women in Ghana. It shows that 36.4 percent of women had experienced at least one form of IPV, with emotional IPV being the most prevalent (31.5%) followed by physical (17.3%) and sexual IPV (7.6%).

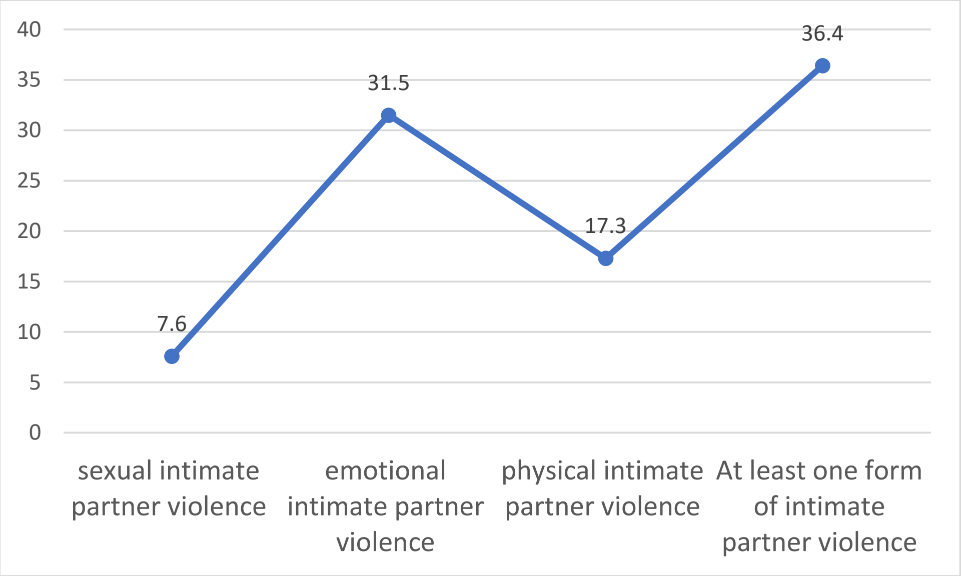

Figure 1: Prevalence of intimate partner violence

Background characteristics and experienced intimate partner violence

Table 1 illustrates that IPV prevalence varies significantly across different demographic and behavioural characteristics. Regarding region of residence, women residing in the Savannah region show the highest IPV proportions of 15.3% sexual IPV, 46.1% emotional IPV, 27.0% physical IPV, and 56.7% experiencing at least one form of IPV. Conversely, the Bono region has the lowest proportions, with 2.0% sexual IPV, 21.7% emotional IPV, 13.4% physical IPV, and 25.3% facing any form of IPV.

Education level also plays a role, with women lacking formal education exhibiting the highest proportions of 8.4% sexual IPV, 35.1% emotional IPV, 23.5% physical IPV, and 41.7% experiencing at least one of the forms of IPV. However, those with higher education levels

report the lowest proportions of 4.1% sexual IPV, 15.5% emotional IPV, 4.7% physical IPV, and 19.2% facing any form of IPV.

Marital status is another significant factor, with women in cohabiting relationships showing the highest proportions, with 10.9% reporting sexual IPV, 37.6% emotional IPV, 20.9% physical IPV, and 42.1% experiencing at least one form of IPV. Contrariwise, married individuals report the lowest proportions of 6.4% sexual IPV, 29.2% emotional IPV, 15.9% physical IPV, and 34.2% facing at least one form of IPV.

Furthermore, women whose partners exhibit domineering behaviour significantly show a higher prevalence of IPV. For instance, women who were often accused of unfaithfulness exhibit the highest proportions of IPV, with 27.0% sexual IPV, 70.7% emotional IPV, 45.9% physical IPV, and 73.2% facing any form of IPV compared to their counterparts who have never experienced these domineering attitudes from their partners.

Table 1: Background characteristics and intimate partner violence

Background characteristics	Frequency N=3,741	Proportion experienced sexual intimate partner violence	Proportion experienced emotional intimate partner violence	Proportion experienced physical intimate partner violence	Proportion experienced at least one form of intimate partner violence
**Demographic**					
Age		X^2=10.5 (p=0.106)	X^2=10.7 (p=0.097)	X^2=7.6 (p=0.269)	X^2=8.7 (p=190)
15 – 19	85	3.7	23.8	23.5	34.1
20 – 24	499	8.2	32.9	20.3	36.7
25 – 29	674	7.4	29.2	13.6	34.5
30 – 34	792	6.7	30.6	15.7	35.0
35 – 39	743	9.4	26.8	15.1	33.5
40 – 44	587	8.3	37.8	21.1	41.4
45 – 49	411	5.6	36.4	20.4	40.2
**Region of residence**		X^2=48.7 (p<0.001)	X^2=94.2 (p<0.001)	X^2=58.1 (p<0.001)	X^2=113.7 (p<0.001)
Western	205	7.6	38.0	18.8	42.4
Central	376	10.1	40.0	23.0	47.0
Greater Accra	536	7.1	29.6	10.6	30.4
Volta	164	9.7	35.0	14.6	37.6
Eastern	285	6.5	31.2	19.0	34.9
Ashanti	722	9.6	28.0	15.6	34.6
Western north	100	5.7	22.4	12.6	25.9
Ahafo	80	8.5	38.4	21.9	48.1

Bono	127	2.0	21.7	13.4	25.3
Bono east	163	5.7	26.4	15.8	30.6
Oti	105	11.2	36.2	21.4	42.4
Northern	384	5.7	34.3	23.2	40.7
Savannah	95	15.3	46.1	27.0	56.7
North east	93	3.5	25.1	16.0	28.4
Upper east	193	3.3	29.1	17.3	32.8
Upper west	112	6.9	22.8	13.4	28.6
Place of residence		X^2=1.2 (p=0.278)	X^2=2.3 (p=0.129)	X^2=4.5 (p<0.05)	X^2=3.6 (p=0.058)
Urban	1,962	8.2	30.2	15.7	34.7
Rural	1,779	7.0	32.9	19.0	38.3
Education		X^2=5.0 (p=0.169)	X^2=40.4 (p<0.001)	X^2=46.8 (p<0.001)	X^2=38.7 (p<0.001)
No education	927	8.4	35.1	23.5	41.7
Primary	565	8.8	40.7	20.8	44.4
Secondary	1,900	7.5	29.9	15.5	34.6
Higher	349	4.1	15.5	4.7	19.2
Wealth status		X^2=8.3 (p=0.080)	X^2=14.5 (p<0.01)	X^2=40.7 (p<0.001)	X^2=19.5 (p<0.01)
Poorest	739	6.2	33.7	21.5	40.0
Poorer	685	7.2	35.0	17.5	39.3
Middle	717	9.0	35.7	20.6	41.3
Richer	816	9.9	30.9	20.0	36.1
Richest	784	5.6	23.2	7.3	26.1
**Marita status**		X^2=29.4 (p<0.001)	X^2=15.8 (p<0.001)	X^2=6.0 (p<0.05)	X^2=11.6 (p<0.01)
Married	2,700	6.4	29.2	15.9	34.2
Cohabitation	1,041	10.9	37.6	20.9	42.1
**Occupation**		X^2=14.8 (p=0.094)	X^2=30.1 (p<0.001)	X^2=26.9 (p<0.001)	X^2=32.9 (p<0.001)
Not working	394	9.9	27.9	17.1	34.2
Professional/technical	248	3.5	20.3	4.5	24.6
Clerical	49	4.0	14.5	1.3	15.1
Sales	360	8.7	31.4	16.6	34.9
Agriculture – self employed	5	0.0	23.5	7.8	27.5
Agriculture - employee	148	4.8	28.6	16.5	32.2
Services	2,110	7.9	34.9	19.7	40.2
Skilled manual	392	6.5	27.5	16.0	31.6
Unskilled manual	17	8.6	22.9	13.5	24.6
Other	18	20.5	38.9	8.9	44.1
**Partner Domineering**					
Husband/partner jealous if respondent talks with other men		X^2=156.0 (p<0.001)	X^2=325.3 (p<0.001)	X^2=262.8 (p<0.001)	X^2=326.0 (p<0.001)
Never	2,340	3.9	21.4	9.8	25.8
Often	514	20.3	57.1	37.6	62.5
Sometimes	771	9.7	42.5	23.6	47.7

Yes, but not in the last 12 months	116	12.2	48.7	35.9	58.8
Husband/partner accuses respondent of unfaithfulness		X^2=191.2 (p<0.001)	X^2=370.0 (p<0.001)	X^2=250.9 (p<0.001)	X^2=338 (p<0.001)
Never	3,079	5.1	24.2	12.6	29.4
Often	247	27.0	70.7	45.9	73.2
Sometimes	350	14.8	60.3	32.4	64.8
Yes, but not in the last 12 months	65	16.8	70.8	47.0	75.0
Husband/partner does not permit respondent to meet female friends		X^2=127.7 (p<0.001)	X^2=208.7 (p<0.001)	X^2=141.5 (p<0.001)	X^2=207.0 (p<0.001)
Never	3,143	5.6	26.9	14.1	31.6
Often	269	21.7	59.0	36.3	62.8
Sometimes	285	15.2	55.9	32.9	64.4
Yes, but not in the last 12 months	44	18.9	33.3	24.1	36.7
Husband/partner tries to limit respondent's contact with family		X^2=156.2 (p<0.001)	X^2=154.1 (p<0.001)	X^2=171.3 (p<0.001)	X^2=154.4 (p<0.001)
Never	3,499	6.3	28.8	15.2	33.6
Often	84	34.4	80.9	57.8	88.4
Sometimes	147	22.9	63.5	39.9	69.9
Yes, but not in the last 12 months	11	14.2	69.0	53.9	76.3
Husband/partner insists on knowing where respondent is		X^2=115.1 (p<0.001)	X^2=250.0 (p<0.001)	X^2=168.7 (p<0.001)	X^2=282.9 (p<0.001)
Never	2,189	3.9	22.3	10.5	26.2
Often	880	15.9	48.3	29.9	54.8
Sometimes	624	8.7	39.5	22.8	45.7
Yes, but not in the last 12 months	48	9.7	37.4	25.0	42.1
**Justification of wife beaten**		X^2=7.1 (p<0.01)	X^2=41.7 (p<0.001)	X^2=113.4 (p<0.001)	X^2=74.5 (p<0.001)
No	2,958	7.2	29.1	13.8	33.0
Yes	783	9.2	40.4	30.2	49.1
Total	3,741	7.6	31.5	17.3	36.4

Binary Logistic Regression analysis of the experience of intimate partner violence

Table 2 shows the binary logistic regression results of the factors associated with different forms of IPV. The results show that region of residence, level of education, partner domineering behaviour, and justification of violence were significantly associated with the experience of IPV. Women who were residing in the Northeast [aOR=0.35, 95%CI=0.17,0.71], Bono [aOR=0.36, 95%CI=0.19,0.65], Upper West [aOR=0.33, 95%CI=0.19,0.56], and Western North [aOR=0.35, 95%CI=0.17,0.71] regions had lower odds experiencing any form of IPV compared to their counterparts in the Western region.

The results demonstrate that increasing levels of women's education have a protective effect on their likelihood of experiencing the three forms of IPV considered in this study. For instance, women with higher levels of education had a reduced risk of 60% of experiencing at least one form of IPV than those with no formal education.

However, women whose partners exhibit domineering behaviours were at a higher risk of experiencing IPV. Thus, women whose partners often get jealous for seeing them talk with other men, accusing them of unfaithfulness, not permitting them to meet female friends, and limiting their contact with family were 1.76, 2.59, 1.59, and 5.75 times, respectively, more likely to experience at least one form of IPV. Similarly, women who justified or endorsed wife beating were more had a higher likelihood [aOR=1.57, 95%CI=1.22,2.02] of experiencing at least one form of IPV than those who did not endorse such behaviour.

Table 2: Binary Logistic Regression and experienced of intimate partner violence

Background characteristics	Sexual intimate partner violence	Emotional intimate partner violence	Physical intimate partner violence	At least one form of intimate partner violence
	A0R (CI)	A0R (CI)	A0R (CI)	A0R (CI)
**Demographic**				
Age				
15 – 19	Ref	Ref	Ref	Ref
20 – 24	2.59(0.90, 7.43)	1.37(0.63, 2.99)	0.70(0.22, 2.23)	0.90 (0.38, 2.17)
25 – 29	2.69(0.93, 7.75)	1.34(0.62, 2.94)	0.45(0.15, 1.35)	0.98(0.41, 2.33)
30 – 34	2.66(0.90, 7.93)	1.75(0.81, 3.79)	0.65(0.23, 1.84)	1.19(0.51, 2.76)
35 – 39	3.95**(1.43, 10.88)	1.45(0.66, 3.19)	0.64(0.21, 1.95)	1.16(0.48, 2.81)
40 – 44	3.43*(1.16, 10.18)	2.41*(1.06, 5.48)	0.92(0.31, 2.70)	1.53(0.64, 3.70)
45 – 49	2.23(0.68, 7.30)	2.43*(1.01, 5.87)	0.93(0.31, 2.83)	1.54(0.59, 4.01)
**Region of residence**				

Western	Ref	Ref	Ref	Ref
Central	0.80(0.35, 1.81)	0.65(0.37, 1.13)	0.68(0.34, 1.33)	0.75(0.44, 1.28)
Greater Accra	0.94(0.45, 1.96)	0.86(0.51, 1.43)	0.61(0.31, 1.18)	0.71(0.44, 1.16)
Volta	1.01(0.47, 2.18)	0.61(0.36, 1.04)	0.52*(0.28, 0.98)	0.57**(0.35, 0.94)
Eastern	0.74(0.33, 1.67)	0.57*(0.33, 0.99)	0.87(0.49, 1.56)	0.60*(0.36, 0.98)
Ashanti	1.11(0.49, 2.49)	0.45**(0.36, 0.77)	0.54*(0.29, 1.00)	0.54**(0.33, 0.90)
Western north	0.85(0.32, 2.21)	0.39**(0.22, 0.69)	0.48*(0.24, 0.97)	0.39**(0.22, 0.69)
Ahafo	1.07(0.48, 2.38)	0.83(0.44, 1.54)	0.91(0.51, 1.63)	1.00(0.58, 1.72)
Bono	0.21*(0.05, 0.78)	0.35**(0.18, 0.68)	0.61(0.28, 1.32)	0.36**(0.19, 0.65)
Bono east	0.91(0.35, 2.37)	0.59(0.34, 1.03)	0.80(0.41, 1.56)	0.59(0.34, 1.02)
Oti	1.47(0.68, 3.16)	0.62(0.34, 1.07)	0.74(0.39, 1.38)	0.64(0.39, 1.07)
Northern	0.57(0.23, 1.43)	0.61(0.33, 1.12)	0.65(0.36, 1.19)	0.60(0.50, 1.77)
Savannah	1.44(0.50, 4.17)	0.78(0.41, 1.49)	0.57(0.31, 1.05)	0.95(0.50, 1.77)
North east	0.41(0.15, 1.09)	0.40*(0.19, 0.84)	0.44(0.19, 1.03)	0.35**(0.17, 0.71)
Upper east	0.50(0.16, 1.55)	0.63(0.31, 1.27)	0.84(0.42, 1.64)	0.59(0.29, 1.19)
Upper west	0.78(0.34, 1.79)	0.32***(0.18, 0.57)	0.33**(0.16, 0.68)	0.33***(0.19, 0.56)
Place of residence				
Urban	Ref	Ref	Ref	Ref
Rural	0.91(0.59, 1.41)	1.00(0.74, 1.35)	1.01(0.71, 1.46)	0.99(0.75, 1.31)
Education				
No education	Ref	Ref	Ref	Ref
Primary	0.70(0.38, 1.28)	1.11(0.69, 1.45)	0.75(0.50, 1.12)	1.01(0.71, 1.44)
Secondary	0.71(0.39, 1.31)	0.85(0.60, 1.20)	0.72(0.50, 1.03)	0.85(0.57, 1.20)
Higher	0.72(0.28, 1.81)	0.39**(0.19, 0.78)	0.51(0.19, 1.35)	0.40**(0.21, 0.77)
Wealth status				
Poorest	Ref	Ref	Ref	Ref
Poorer	0.86(0.49, 1.50)	1.00(0.69, 1.45)	0.71(0.49, 1.03)	1.13(0.86, 1.48)
Middle	1.18(0.65, 2.15)	0.99(0.65, 1.50)	0.93(0.56, 1.53)	0.97(0.64, 1.45)
Richer	1.51(0.83, 2.76)	0.91(0.56, 1.47)	1.29(0.76, 2.19)	0.91(0.58, 1.44)
Richest	1.09(0.49, 2.45)	0.75(0.41, 1.34)	0.54(0.27, 1.11)	0.69(0.39, 1.21)
**Marita status**				
Married	Ref	Ref	Ref	Ref
Cohabitation	1.27(0.83, 1.97)	1.22(0.91, 1.63)	1.13(0.79, 1.60)	1.13(0.86, 1.48)
**Occupation**				
Not working	Ref	Ref	Ref	Ref
Professional/technical	0.35*(0.13, 0.98)	1.70(0.84, 3.44)	0.54(0.21, 1.39)	1.52(0.80, 2.93)
Clerical	0.45(0.14, 1.28)	0.86(0.30, 2.41)	0.12*(0.01, 0.98)	0.61(0.23, 1.66)
Sales	0.75(0.35, 1.59)	1.01(0.62, 1.64)	0.99(0.56, 1.74)	0.94(0.59, 1.50)
Agriculture – self employed	1	0.44(0.08, 2.47)	0.32(0.05, 1.92)	0.37(0.19, 1.61)
Agriculture - employee	0.48(0.18, 1.32)	0.83(0.50, 1.42)	0.69(0.37, 1.27)	0.72(0.43, 1.18)
Services	0.66(0.36, 1.19)	1.27(0.88, 1.84)	1.21(0.78, 1.89)	1.21(0.85, 1.71)
Skilled manual	0.59(0.29, 1.24)	1.08(0.67, 1.76)	1.14(0.62, 2.09)	0.95(0.59, 1.54)
Unskilled manual	1.04(0.18, 6.10)	0.82(0.33, 2.04)	0.89(0.15, 5.29)	0.64(0.28, 1.50)
Other	3.56(0.68, 18.48)	1.67(0.40, 7.04)	0.54(0.10, 2.73)	1.69(0.42, 6.84)

**Partner Domineering**				
Husband/partner jealous if respondent talks with other men				
Never	Ref	Ref	Ref	Ref
Often	1.94(1.00, 3.76)	1.80**(1.28, 2.54)	2.46***(1.62, 3.73)	1.76**(1.25, 2.48)
Sometimes	1.69(0.96, 3.00)	1.72***(1.31, 2.26)	1.94***(1.37, 2.75)	1.66***(1.29, 2.13)
Yes, but not in the last 12 months	2.37(0.81, 7.00)	2.47**(1.27, 4.80)	4.37**(1.89, 10.12)	3.20***(1.71, 5.98)
Husband/partner accuses respondent of unfaithfulness				
Never	Ref	Ref	Ref	Ref
Often	2.21*(1.04, 4.72)	3.23***(1.91, 5.45)	2.11**(1.19, 3.74)	2.59***(1.03, 2.46)
Sometimes	1.60(0.86, 2.97)	2.70***(1.96, 3.73)	1.62*(1.10, 2.38)	2.42***(1.22, 2.97)
Yes, but not in the last 12 months	2.29(0.82, 6.38)	5.53***(2.45, 12.52)	3.40(0.99, 11.66)	4.93***(2.18, 11.42)
Husband/partner does not permit respondent to meet female friends				
Never	Ref	Ref	Ref	Ref
Often	1.60(0.85, 3.03)	1.75*(1.13, 2.72)	1.41(0.88, 2.26)	1.59*(1.03, 2.46)
Sometimes	1.36(0.73, 2.52)	1.62*(1.01, 2.61)	1.24(0.79, 1.95)	1.90**(1.22, 2.97)
Yes, but not in the last 12 months	1.97(0.78, 4.97)	0.55(0.21, 1.41)	0.73(0.28, 1.88)	0.45(0.18, 1.12)
Husband/partner tries to limit respondent's contact with family				
Never	Ref	Ref	Ref	Ref
Often	2.73*(1.24, 6.02)	3.56**(1.60, 7.92)	2.67*(1.14, 6.27)	5.75***(2.27, 13.42)
Sometimes	2.55*(1.22, 5.34)	2.13**(1.27, 3.58)	1.97*(1.10, 3.53)	2.32**(1.35, 3.99)
Yes, but not in the last 12 months	1.46(0.34, 6.37)	2.52(0.69, 9.12)	2.91(0.69, 12.28)	3.01(0.90, 10.07)
Husband/partner insists on knowing where respondent is				
Never	Ref	Ref	Ref	Ref
Often	1.98**(1.20, 3.27)	1.55**(1.60, 7.92)	1.78**(1.22, 2.58)	1.70***(1.27, 2.29)
Sometimes	1.37(0.83, 2.27)	1.34*(1.02, 1.76)	1.40(0.96, 2.04)	1.41**(1.07, 1.86)
Yes, but not in the last 12 months	0.77(0.25, 2.34)	1.25(0.54, 2.87)	1.18(0.52, 2.65)	1.19(0.53, 2.70)
**Justification of wife beaten**				

No	Ref	Ref	Ref	Ref
Yes	1.12(0.68, 1.84)	1.33*(1.00, 1.77)	2.31***(1.66, 3.20)	1.57***(1.22, 2.02)

***p<0.001, **p<0.01,*p<0.05 Ref=Reference category

A0R= Adjusted Odds Ratio, CI=Confidence interval

Discussion

This study aims to provide current insight into women's experience of IPV in Ghana. Consistent with the global [1] and regional [2] estimates, we found that 36.4 percent of women had ever experienced one form of IPV. Additionally, our result aligns with previous studies from Pakistan [17] and Kenya [18] that have found emotional violence to be the most common IPV type experienced. The observed pattern of the IPV typologies is expected. Sexual violence often comes with heightened fear and shame which tends to discourage victims from reporting such acts. This assertion is corroborated by Orchowski et al. [19] who argue that victims of sexual violence do not report due to factors such as *"labeling of the experience, age, fear, privacy concerns, self-blame, betrayal/shock, the relation/power of the perpetrator, negative reactions to disclosure, and the belief—or personal experience—that reporting would not result in justice"*. As such, the low prevalence of sexual violence reported in this study may not be the true reflection of the situation but rather a reflection of the inability of women to open up about their experiences.

Regarding the associated factors, our study revealed that educational attainment, partner domineering behaviour and justification of violence were the only factors that significantly predicted the women's likelihood of experiencing at least one form of IPV. Specifically, women with higher educational attainment had a significantly lower likelihood of being victims of IPV. The result aligns with Iqbal and Fatmi [17] who found low educational attainment to be a high risk factor for IPV victimization. Our finding is corroborated by Weitzman [20] study which revealed that increasing women's schooling significantly reduces their risk of becoming victims of emotional, physical and sexual IPV. This association is best explained from the social causation perspective which postulates that an individual's socioeconomic status is the cause of their weakened mental health [21]. In the context of IPV, it implies that women with higher levels of education may have better access to resources, social support networks, and coping strategies, which in turn reduce their vulnerability to IPV.

Furthermore, education is often linked to higher social status and exposure to progressive attitudes toward gender roles and interpersonal relationships. Women with higher education levels may be less likely to adhere to traditional gender norms that justify or tolerate violence against women. They may have more egalitarian relationships characterized by mutual respect and non-violent conflict resolution strategies.

Our study shows that women whose partners exhibited domineering attitudes (i.e., getting jealous, not permitting women to meet female friends, partner limiting woman's contact with her family, etc.) were more likely to experience at least one form of IPV. This association resonates with Capaldi et al. [22] who mapped literature and found possessiveness and jealousy on the part of male partners exacerbated the risk of IPV among women. According to Aizpurua et al. [23], partner domineering or controlling behaviours tend to generate fear in women and adversely impact their self-esteem. This diminished self-esteem may impede women from recognizing and addressing subtle forms of violence, which can escalate into more severe instances of IPV. Therefore, the association observed in our study underscores the importance of addressing and intervening in domineering behaviours within intimate relationships to mitigate the risk of IPV and safeguard the well-being of women.

We found that women who justified or endorsed wife beating were at a 57 percent higher risk of becoming victims of at least one form of IPV. Our result is in contrast to a Nepali study [24] that found no significant association between acceptance of wife beating and life-time or past year physical IPV experience among women. A possible explanation for this association could be that women who endorse such attitudes may be more likely to accept and internalize abusive behaviours within their relationships, creating a conducive environment for IPV to occur. There were also significant differences in the risk of IPV by region; thus, underscoring a need for more region-specific tailored interventions to address IPV among women.

Strengths and limitations

We relied on the 2022 GDHS which provides a closer current picture of IPV among women of reproductive age in Ghana. This ensures that our findings reflect the present status quo. Also, the GDHS data collection follows systematic methodologies and a sampling process which ensures that the findings are representative at the district and national levels. Nonetheless,

the estimated prevalence of IPV may be underestimated given that the data is self-reported. Also, we are unable to establish a causal association between educational attainment, partner domineering behaviour, and the endorsement of violence as significant predictors of IPV since the GDHS is based on a cross-sectional design.

Conclusion

In conclusion, the study's identification of educational attainment, partner domineering behaviour, and the endorsement of violence as significant predictors of IPV highlights areas for targeted intervention. Policies aimed at promoting education, particularly for women, not only contribute to their economic empowerment but also serve as a protective factor against IPV. Furthermore, addressing partner domineering behaviour through educational campaigns, counselling services, and legal frameworks can help empower women and create safer relationship dynamics. Efforts to challenge cultural attitudes that normalize or justify violence, such as the endorsement of wife beating, are essential for shifting societal norms and fostering environments where IPV is not tolerated.

Abbreviations

AOR: Adjusted Odds Ratio; GDHS: Ghana Demographic and Health Survey; IPV: Intimate Partner Violence; Ref: Reference Category; SSA: Sub-Saharan Africa; WHO: World Health Organization

Declarations

Ethical approval and consent to participate

We did not need to seek ethical clearance because the DHS dataset we used is publicly available. We obtained the datasets from the DHS Program after completing the necessary registration and getting approval for their use. We followed all the ethical guidelines that pertain to using secondary datasets in research publications. You can find detailed information about how we used the DHS data and the ethical standards we followed at this link: http://goo.gl/ny8T6X.

Consent for publication

None declared

Availability of data and materials

The datasets generated and/or analysed during the current study are available in the Measure DHS repository: http://dhsprogram.com/data/available-datasets.cfm.

Competing interests

None

Funding

We received no funding.

Authors' contributions

KSD: Conceptualization; Data curation; Formal analysis; Methodology; Software; Writing – original draft; Writing – review & editing

CA: Conceptualization; Methodology; Supervision; Validation; Writing – original draft; Writing – review & editing

JO: Conceptualization; Methodology; Supervision; Validation; Writing – original draft; Writing – review & editing

Acknowledgements

We acknowledge the Measure DHS for granting us free access to the dataset used in this study.

References

1. Seidu AA, Dzantor S, Sambah F, Ahinkorah BO, Ameyaw EK. Participation in household decision making and justification of wife beating: evidence from the 2018 Mali Demographic and Health Survey. International health. 2022 Jan;14(1):74-83.
2. World Health Organization (WHO). Violence against women: key facts. March 2021. Available at: https://www.who.int/news-room/fact-sheets/detail/violence-against-women.

3. World Health Organization. *Global and regional estimates of violence against women: prevalence and health effects of intimate partner violence and non-partner sexual violence*. Available from: https://www.who.int/publications/i/item/9789241564625.
4. Adu C. Socio-economic inequalities in intimate partner violence justification among women in Ghana: analysis of the 2014 Ghana Demographic and Health Survey data. International health. 2023 Mar;15(2):182-8.
5. Aboagye, R.G., Donkoh, I.E., Okyere, J. et al. Association between sexual violence and multiple high-risk fertility behaviours among women of reproductive age in sub-Saharan Africa. BMC Public Health . 2024 Feb;24, 432.
6. World Health Organization (WHO). Intimate partner violence: Violence info. Available at: https://apps.who.int/violence-info/intimate-partner-violence/.
7. Maxwell L, Nandi A, Benedetti A, Devries K, Wagman J, García-Moreno C. Intimate partner violence and pregnancy spacing: results from a meta-analysis of individual participant time-to-event data from 29 low-and-middle-income countries. BMJ Glob Health. 2018;3(1):e000304.
8. Liu X, Logan J, Alhusen J. Cardiovascular risk and outcomes in women who have experienced intimate partner violence: an integrative review. Journal of cardiovascular nursing. 2020 Jul 1;35(4):400-14.
9. Ola BE. What factors are associated with recent changes in men's attitudes towards intimate partner violence across regional, rural, and urban spaces of Ghana? Findings from three waves of Ghana national surveys from 2003 to 2014. Journal of interpersonal violence. 2022 May;37(9-10):NP8190-225.
10. Cofie N. A multilevel analysis of contextual risk factors for intimate partner violence in Ghana. International review of victimology. 2020 Jan;26(1):50-78.
11. Sedziafa AP, Tenkorang EY, Owusu AY. Kinship and intimate partner violence against married women in Ghana: A qualitative exploration. Journal of interpersonal violence. 2018 Jul;33(14):2197-224.
12. Rohn E, Tenkorang EY. Structural and institutional barriers to help-seeking among female victims of intimate Partner violence in Ghana. Journal of family violence. 2023 Jul;38(5):815-27.
13. Corsi DJ, Neuman M, Finlay JE, Subramanian SV. Demographic and health surveys: a profile. International journal of epidemiology. 2012 Dec 1;41(6):1602-13.

14. Ghana Statistical Service (GSS), Ghana Health Service (GHS), ICF International. Ghana Demographic and Health Survey 2022. 2023; Rockville, MD: GSS, GHS, and ICF International. https://www.dhsprogram.com/pubs/pdf/FR387/FR387.pdf.
15. Ahinkorah BO, Aboagye RG, Cadri A, Salihu T, Seidu AA, Yaya S. Exposure to interparental violence and intimate partner violence among women in Papua New Guinea. BMC women's health. 2023 Feb 7;23(1):48.
16. Dickson KS, Boateng EN, Adzrago D, Addo IY, Acquah E, Nyarko SH. Silent suffering: unveiling factors associated with women's inability to seek help for intimate partner violence in sub-Saharan Africa (SSA). Reproductive health. 2023 Jul 26;20(1):110.
17. Iqbal M, Fatmi Z. Prevalence of emotional and physical intimate partner violence among married women in Pakistan. Journal of interpersonal violence. 2021 May;36(9-10):NP4998-5013.
18. Ringwald B, Tolhurst R, Taegtmeyer M, Digolo L, Gichuna G, Gaitho MM, Phillips–Howard PA, Otiso L, Giorgi E. Intra-urban variation of intimate partner violence against women and men in Kenya: Evidence from the 2014 Kenya Demographic and Health Survey. Journal of interpersonal violence. 2023 Mar;38(5-6):5111-38.
19. Orchowski LM, Grocott L, Bogen KW, Ilegbusi A, Amstadter AB, Nugent NR. Barriers to reporting sexual violence: a qualitative analysis of# WhyIDidntReport. Violence against women. 2022 Nov;28(14):3530-53.
20. Weitzman A. Does increasing women's education reduce their risk of intimate partner violence? Evidence from an education policy reform. Criminology. 2018 Aug;56(3):574-607.
21. Kröger H, Pakpahan E, Hoffmann R. What causes health inequality? A systematic review on the relative importance of social causation and health selection. The European Journal of Public Health. 2015 Dec 1;25(6):951-60.
22. Capaldi DM, Knoble NB, Shortt JW, Kim HK. A systematic review of risk factors for intimate partner violence. Partner abuse. 2012 Jan 1;3(2):231-80.
23. Aizpurua E, Copp J, Ricarte JJ, Vázquez D. Controlling behaviors and intimate partner violence among women in Spain: An examination of individual, partner, and relationship risk factors for physical and psychological abuse. Journal of interpersonal violence. 2021 Jan;36(1-2):231-54.

24. Yoshikawa K, Shakya TM, Poudel KC, Jimba M. Acceptance of wife beating and its association with physical violence towards women in Nepal: a cross-sectional study using couple's data. PloS one. 2014 Apr 21;9(4):e95829.
